# Trends in complementary feeding practices and caregivers’ feeding knowledge among children aged 6-23 months: Repeated cross-sectional surveys in rural Qinghai China 2012-18

**DOI:** 10.7189/jogh.11.08003

**Published:** 2021-04-03

**Authors:** Qiong Wu, Yiwen Huang, Michelle Helena van Velthoven, Wei Wang, Yanfeng Zhang

**Affiliations:** 1 Department of Integrated Early Childhood Development, Capital Institute of Pediatrics, Beijing, China; 2 Nuffield Department of Primary Care Health Sciences, University of Oxford, Oxford, UK

## Abstract

**Background:**

Appropriate infant and young child feeding is the basis for child survival, growth and development. The aim of this study was to investigate trends in complementary feeding practices and caregivers’ feeding knowledge among children from 2012 to 2018 in Huzhu County, Qinghai Province, China.

**Methods:**

This study took place during and after a controlled interventional evaluation trial in Qinghai Province, China, which aimed to evaluate the effectiveness of community-based nutrient-dense complementary food supplements (YingYangBao) combined with dietary counseling on improving 6-23 month-old children's health status. We conducted four representative cross-sectional surveys on caregivers of children aged 6-23 months in Huzhu County, Qinghai Province, China (baseline survey for the trial (N = 1804) in August 2012, end-line survey for the trial (N = 2186) in August 2014, follow-up survey 1 (N = 496) in January 2016, and follow-up survey 2 (N = 754) in July 2018). In all surveys we used the same questionnaire to collect household information, infant feeding practices and caregivers’ feeding knowledge.

**Results:**

During the trial period (2012-2014), the proportion of children aged 6-8 months that introduced (semi-) solid food increased from 86.1% to 96.3% (*P* < 0.0001), however, there was a downward trend from 2014 to 2018 (*P* = 0.0014 for trend). The prevalence of minimum dietary diversity also increased from 51.4% at 2012 baseline survey to 57.5% at 2014 endline survey (*P* = 0.0004), but the upward trend did not maintain from 2014 to 2018 (*P* = 0.7863 for trend). The minimum dietary frequency, the minimum acceptable diet, and continued breastfeeding at one year were nearly unchanged from 2012 to 2018 (*P* = 0.9529, *P* = 0.7602 and *P* = 0.6013 for trend, respectively), remaining around 30%, 10% and 20% respectively in the four surveys. Caregivers’ feeding knowledge on the duration of exclusive breastfeeding and introduction of semi or solid foods at 6-8 months increased from 2012 to 2018 (18.6% to 39.5%, 43.2% to 64.3%, respectively).

**Conclusion:**

This study showed that the sustainability of community-based YYB and dietary counseling program was suboptimal. We suggest that multiple information delivery channels such as smartphones and the Internet should be explored as a supplement to existing channels for delivering counseling information.

**Trial registration:**

ChiCTRPRC12002444.

Although China has made rapid economic growth during the past two decades, a large number of children still suffer from malnutrition. It is reported that the prevalence of stunting and underweight for children aged below five years in 2013 was 8.2% and 4.9%, respectively. This translates to an estimated seven-million Chinese children being stunted and two-million being underweight [1,2]. Moreover, anemia prevalence in children aged 6-12 months and 13-24 months in rural China was 28.2% and 20.5% in 2010, respectively [3].

Appropriate infant and young child feeding is the basis of good nutrition which promotes healthy growth and development and lowers the risk of disease in later life [4-6]. Furthermore, the period of complementary feeding (6-24 months) is well recognized as one of the most critical times for preventing malnutrition [5]. Growth faltering and micronutrient deficiencies are highly prevalent among infants and young children at this period [5]. The Global Strategy for Infant and Young Child Feeding, developed by the World Health Organization (WHO) and United Nations Children’s Fund (UNICEF), recommend that infants should be exclusively breastfed for six months after birth, and be introduced complementary foods after six months with continued breastfeeding until two years or above [7]. However, the infant and young child feeding practices were not optimal in China. Chinese national data in 2013 showed that the exclusive breastfeeding rate under six months was only 18.6% [8] and the prevalence of minimum dietary diversity, meal frequency and acceptable diet among children aged 6-23 months was 53.7%, 69.1%, and 25.1%, respectively [8]. Studies also indicate that complementary foods generally contained mainly carbohydrates and lacked protein and fat [8], or were introduced to children too early or too late. Also, they were given in too small amounts or not frequently enough [9,10].

During the past decade, China made lots of efforts to promote infant feeding and Child nutrition. Since 2009, China has been implementing a national program “Basic Public Health Service”, in which health care workers are required to provide face-to-face breastfeeding and complementary feeding counseling to pregnant women and mothers throughout antenatal and postnatal care [11]. Moreover, Chinese government initiated a national community-based nutritional program to improve children's nutrition in poor rural areas in 2011, which provided free nutrient-dense complementary food supplement for infants and young children (“Ying Yang Bao”, YYB) to all children aged 6-23 months in poor rural areas [12]; and the program had covered 823 poor counties by 2019 in China [13].

From 2012 to 2014, our team carried out a controlled interventional evaluation trial in Qinghai Province, China to evaluate the effectiveness of YYB combined with dietary counseling on improving 6-23 months old children's health status, improving complementary feeding practices (introduced to (semi-) solid food at 6-8 months, minimum dietary diversity) and decreased anemia prevalence [14,15]. Although the evaluation trial ended, the local government continued implementing the community-based YYB program. Therefore, we conducted another two follow-up assessments to monitor the program implementation in the intervention county in 2016 and 2018 respectively. Current paper is based on the trial data and follow-up data in this study from 2012 to 2018. The aim of this paper was to explore the time trend in complementary feeding practices and caregivers’ feeding knowledge in rural areas Qinghai, which could help local government understand the sustainability of the program in this area, and provide constructive suggestions to policymakers.

## METHODS

### Study design

This study took place during and after a controlled interventional evaluation trial in Qinghai Province, China, which aimed to evaluate the effectiveness of YYB combined with dietary counseling on improving 6-23 months children's health status. More details of the controlled interventional study can be found in our previously published papers [14,15]. We conducted four cross-sectional surveys with caregivers of children aged 6-23 months in the intervention county at the baseline survey for the trial in August 2012, end-line survey for the trial in August 2014, follow-up survey 1 in January 2016 and follow-up survey 2 in July 2018. All surveys used the same questionnaire to collect household information, infant feeding practices, and caregivers’ feeding knowledge.

### Study setting

Qinghai Province lies in northwest China, with an area of around 720 000 km^2^.The resident population of the province was 6.0782 million by the end of 2019 [16]. Qinghai Province has 34 counties and 439 townships. The Qinghai resident per capita disposable income in 2019 was ¥11499 (US$1714.94) for rural people [16], which was far lower than the national level (¥16021; US$2389.48)) [17].

Huzhu County is located in the northeastern part of Qinghai Province. The county has 19 townships and 294 villages, with a total population of 401 540, of which the rural population accounted for 76.0%. The rural resident per capita disposable income in Huzhu County was ¥11760 (US$1753.21) [18]. Huzhu County is the intervention county of the controlled interventional evaluation trial.

### Participants

Children aged 6-23 months and their caregivers in Huzhu County were invited to participate in all four surveys. We excluded children with a structural or genetic birth defect such as neural tube defects, congenital heart disease or phenylketonuria or caregivers who refused to participate.

### Study instrument

We used the questionnaire, the maternal, newborn and child health household survey (MNCH HHS) questionnaire [19], to collect household information, infant feeding practices and children’s consumption of YYB in each sampled village in all four surveys.

### Sample size and sampling

The sample size required for the trial’s baseline and end-line surveys were calculated based on previously collected data (17.2% for stunting and 44.2% for anemia for children aged 6-23 months, respectively, unpublished data). With a 20% reduction in the prevalence of stunting and anemia being expected, a sample size of 1793 children aged 6-23 months was sufficient for all key indicators for both surveys. The two-stage sampling procedure was used to select children in the 2012 baseline survey and the 2014 end-line survey. At the first stage, we selected 150 villages out of 294 villages using proportional to population size (PPS) sampling method. At the second stage, we obtained the name list of all eligible children aged 6-23 months in each selected village from the local maternal and child health family planning service center, and then randomly selected and interviewed 12 children and their caregivers per village in the 2012 baseline survey and 15 children and their caregivers per village in the 2014 end-line survey. The details were reported in the effectiveness of the controlled interventional study paper [14].

The sample size required for the two follow-ups were calculated based upon baseline data. The main goal of follow-ups was to evaluate the effectiveness of YYB combined with dietary counseling on the prevalence of anemia. We expected to achieve a 20%-point reduction in anemia prevalence and 20%-50% points increase for knowledge and practices of appropriate feeding. With 80% power and 5% significance level, we calculated that the sample size of 458 children aged 6-23 months would be sufficient for all key indicators for follow-ups. We over-sampled with 10% to compensate for possible refusal and loss to follow-up.

The two-stage sampling procedure was also used to select children surveyed in the first follow-up in 2016. To maintain the consistency of the assessment, at the first stage, we randomly selected 40 villages from the 150 villages that were selected and surveyed in the 2012 baseline survey. At the second stage, we randomly selected and interviewed 12 children and their caregivers from the name list of all eligible children in each selected village. While in the second follow-up in 2018, we surveyed all eligible children aged 6-23 months and their caregivers in the same 40 sampling villages as in the first follow-up survey.

### Training of interviewers

Staff from the Capital Institute of Pediatrics in Beijing were supervisors in all four surveys, and students recruited from the School of Public Health, Qinghai University (50 for baseline, 35 for end-line and 25 for the follow-up in 2016) and Qinghai Institute of Health Sciences (21 for the follow-up in 2018) collected data from participants. We provided them training for two days before fieldwork, which included communication skills, explanation of questionnaires, demonstration, role plays, field practice, and group discussions. After the training, a half-day field practice was held in a village clinic. Any problems arising during the field practice were discussed and solved directly.

### Data collection

We collected the baseline data and end-line data in August 2012 and August 2014, respectively. The two follow-ups were conducted in January 2016 and in July 2018, respectively. In each survey, every surveyed township staff notified the village doctors of the sample village in advance, then the village doctors called the caregivers to take their children to the village clinics. Before the survey, interviewers first introduced the aim of the survey to the mothers or other caregivers and obtained written informed consent from them. Then the interviewers questioned them following the instructions of the questionnaire.

In the first follow-up, we used paper-based household survey questionnaires, and all data were entered by two people separately with EpiData 3.1 (EpiData Software, Odense, Denmark). The two files were compared, and discrepancies resolved by referring to the original records. For the other three surveys, we used smartphones with the household survey questionnaire set up in specially developed software to record data [19]. Data for each questionnaire were uploaded into an Excel database via the Internet server.

### Data analysis

Questionnaire data was captured and pooled into a Microsoft Excel sheet. After the data cleaning, we converted the database into a database file (dbf) for the final analysis.

We carried out statistical analysis with SAS 9.2 for Windows (Statistical Analysis System Inc, Cary, NC, USA). The median (interquartile range) was used to describe in years the age of mothers and grandparents of children. Percentages were used to present in categorical variables, including feeding practices, feeding knowledge and information sources. We used the Pearson χ^2^ test and Fisher exact test to compare categorical variables.

Five core complementary feeding practice indicators were calculated according to the WHO guidelines ‘Indicators for assessing infant and young child feeding practices’ [20], including 1) introduction of solid, semi-solid or soft foods, 2) minimum dietary diversity, 3) minimum meal frequency, 4) minimum acceptable diet, 5)consumption of iron-rich or iron-fortified foods. ‘Consumption of meat’ was defined as ‘Proportion of children 6-23 months of age who receive any meat, containing organ meat and fresh or dried fish’. As the age distribution of surveyed children differed among the four surveys, we adjusted the feeding practice indicators by using the total number of children in the four surveys as the standard population.

Moreover, we carried out χ^2^ test for trend analysis to explore the time trend of complementary feeding practice indicators from 2012 to 2018 (baseline to the second follow-up), and 2014 to 2018 (endline to the second follow-up), respectively, and considered two-tailed *P* values of <0.05 for a significant difference.

### Ethical considerations

The study was approved by the Ethics Committee of the Capital Institute of Pediatrics, Beijing, China. All interviewees read the Information Sheet and provided written consent.

**Trial registration number:** ChiCTRPRC12002444.

## RESULTS

A total of 1804 children aged 6-23 months and their caregivers in Huzhu County were surveyed in the 2012 baseline survey, 2186 in the 2014 end-line survey, 496 in the 2016 and 754 in the 2018 follow-up surveys. All the caregivers agreed to participate in the surveys. Characteristics of surveyed children and their caregivers for the four surveys are shown in **Table 1**. Nearly all main caregivers of the children surveyed were mothers and grandparents, and about 70% of mothers were Han nationality, followed by Tu and Tibetan nationality. More than half of the mothers attended junior high school, and the proportion of mothers who were illiterate continuously decreased from 14.5% in the baseline survey to 4.0% in the follow-up 2018 (4.0%). Although the proportion of illiteracy of grandparents decreased as well, more than 60% of them were still illiterate. The main source of household income was working outside the county, followed by agriculture-related work such as growing crops/vegetables and animal husbandry.

**Table1:** Characteristics of surveyed children and their caregivers

Surveys	Baseline survey(N = 1804)	End-line survey(N = 2186)	Follow-up 2016(N = 496)	Follow-up 2018(N = 754)
**Children**				
Age, % (n):				
6-11months	33.8 (610)	35.5 (775)	29.2 (145)	32.8 (247)
12-17months	26.8 (484)	29.0 (635)	38.1 (189)	32.6 (246)
18-23months	39.4 (710)	35.5 (776)	32.7 (162)	34.6 (261)
Sex, % (n):
Male	53.2 (960)	54.8 (1198)	53.5 (254)*	52.1 (393)
Female	46.8 (844)	45.2 (988)	46.5 (221)*	47.9 (361)
**Main caregivers**				
Mother	53.2 (960)	51.8 (1131)†	59.3 (291)‡	48.7 (367)
Grandparent	45.0 (812)	47.8 (1045)†	37.3 (183)‡	46.4 (350)
Father	0.6 (11)	0.2 (4)†	2.4 (12)‡	4.8 (36)
Other	1.2 (21)	0.2 (5)†	1.0 (5)‡	0.1 (1)
**Mothers**				
Age in years (median, interquartile range)	26 (23, 30)	27 (24, 31)	28 (25, 31)§	29 (26, 31)
Nationality, % (n):
Han	68.2 (1228)	70.4 (1526)	74.9 (350)	69.9 (523)
Tu	17.9 (323)	18.4 (398)	16.1 (75)	22.8 (171)
Tibetan	11.1 (200)	10.2 (222)	7.5 (35)	6.3 (47)
Hui	2.6 (46)	0.5 (11)	0.6 (3)	0.3 (2)
Others	0.2 (4)	0.5 (11)	0.9 (4)	0.7 (5)
Education, % (n):
Illiterate	14.5 (261)	11.4 (248)	9.4 (44)	4.0 (30)
Primary school	23.1 (416)	17.1 (371)	20.4 (95)	13.8 (103)
Junior high school	51.3 (925)	54.1 (1172)	55.9 (261)	61.6 (461)
Senior high school or above	8.4 (151)	11.9 (257)	12.6 (59)	17.4 (130)
Do not know	2.7 (48)	5.5 (120)	1.7 (8)	3.2 (24)
**Grandparents**				
Age in year (median, interquartile range)	51 (47, 57)	51 (48, 56)	52 (49,56)‖	54 (50, 59)
Education, % (n):				
Illiterate	69.1 (561)	69.0 (721)	62.8 (115)	60.3 (211)
Primary school	18.1 (147)	18.3 (191)	18.0 (33)	22.6 (79)
Junior high school	11.0 (89)	11.2 (117)	16.4 (30)	15.1 (53)
Senior high school	1.1 (9)	1.0 (11)	2.2 (4)	1.1 (4)
Do not know	0.7 (6)	0.5 (5)	0.6 (1)	0.9 (3)
**Household income**				
Working outside the county	67.1 (1210)	80.8 (1766)**	74.5 (362)††	89.2 (673)
Agriculture-related work	28.1 (507)	14.5 (317)**	19.6 (95)††	6.4 (48)
Self-employed	3.2 (57)	3.8 (84)**	4.7 (23)††	2.9 (22)
Others	1.3 (24)	0.7 (15)**	1.2 (6)††	1.2 (9)
Do not know	0.3 (6)	0.2 (3)**	0.0 (0)††	0.3 (2)

*Twenty-one interviewees were missing for this calculation.

†One interviewee was missing for this calculation.

‡Five interviewees were missing for this calculation.

§Two interviewees were missing for this calculation.

‖Three interviewees were missing for this calculation.

**One interviewee was missing for this calculation.

††Ten interviewees were missing for this calculation.

### Infant feeding practices

**Figure 1:**
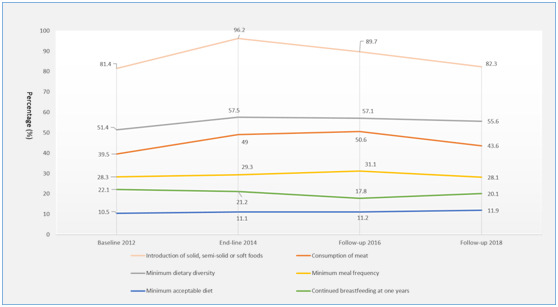
Trends in complementary feeding practices from 2012 to 2018. **Panel A.** Introduction of solid, semi-solid or soft foods: baseline 2012 vs endline 2014, *P* < 0.0001; trend *P* = 0.7979 from 2012 to 2018; trend *P* = 0.0014 from 2014 to 2018. **Panel B.** Minimum dietary diversity: baseline 2012 vs endline 2014, *P* = 0.0004; trend *P* = 0.5831 from 2012 to 2018; trend *P* = 0.7863 from 2014 to 2018. **Panel C.** Consumption of meat: baseline 2012 vs endline 2014, *P* < 0.0001; trend *P* = 0.5327 from 2012 to 2018; trend *P* = 0.4446 from 2014 to 2018. **Panel D.** Minimum meal frequency: baseline 2012 vs endline 2014, *P* = 0.2753; trend *P* = 0.9529 from 2012 to 2018; trend *P* = 0.8524 from 2014 to 2018. **Panel E.** Continued breastfeeding at one year: baseline 2012 vs endline 2014, *P* = 0.7642; trend *P* = 0.6013 from 2012 to 2018; trend *P* = 0.8450 from 2014 to 2018. **Panel F.** Minimum acceptable diet: baseline 2012 vs endline 2014, *P* = 0.4267; trend *P* = 0.7602 from 2012 to 2018; trend *P* = 0.8587 from 2014 to 2018.

Infant feeding practices in the county were suboptimal (**Figure 1**). Although the proportion of children achieving the recommended minimum dietary diversity increased from 51.4% at the 2012 baseline to 57.5% at the 2014 endline (*P* = 0.0004), there had no upward trend after then (*P* = 0.7863 for trend). Furthermore, the minimum dietary frequency, the minimum acceptable diet, and continued breastfeeding at one year were nearly unchanged from 2012 to 2018 (*P* = 0.9529, *P* = 0.7602 and *P* = 0.6013 for trend, respectively), remaining around 30%, 10% and 20% respectively in the four surveys. The proportion of consumption of meat increased to 49% at 2014 endline survey and increased to 50.6% at the 2016 follow-up survey, which was significantly higher than that at 2012 baseline survey (39.6%, *P* <0.0001 and *P* < 0.0001, respectively); the time trend was not significant (*P* = 0.5327 for trend). The proportion of children aged 6-8 months that were introduced to solid, semi-solid or soft foods increased from 81.4% at the 2012 baseline to 96.2% at the 2014 endline (*P* < 0.0001), however, the proportion decreased to 82.3% at 2018 follow-up, which showed a significant downward trend (*P* = 0.0014 for trend).

### Caregivers’ feeding knowledge

**Figure 2:**
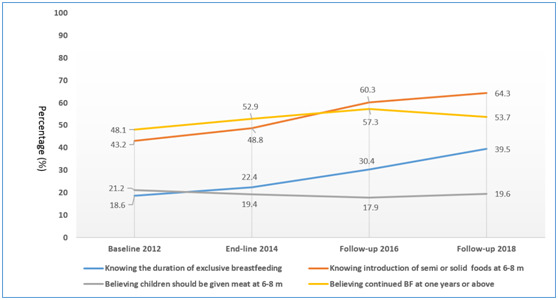
Trends in caregivers’ feeding knowledge from 2012 to 2018. **Panel A.** Knowing the duration of exclusive breastfeeding: trend *P* = 0.004 from 2012 to 2018. **Panel B.** Knowing introduction of semi or solid foods at 6-8 months: trend *P* = 0.0008 from 2012 to 2018. **Panel C.** Believing children should be given meat at 6-8 months: trend *P* = 0.7223 from 2012 to 2018. **Panel D.** Believing continued BF at one years or above: trend *P* = 0.1711 from 2012 to 2018.

As shown in **Figure 2**, the overall knowledge of caregivers about feeding recommendations in four surveys were suboptimal as well. From 2012 to 2018, the estimated percentage of caregivers knowing the duration of exclusive breastfeeding increased from 18.6% to 39.5% (difference, 20.89%, 95% CI = 19.31-22.47; *P* = 0.0004 for trend). The estimated percentage of caregivers knowing introduction of semi or solid foods at 6-8 months increased from 43.2% to 64.3% (difference, 21.14%, 95% CI = 19.56-22.72; *P* = 0.0008 for trend). However, the estimated percentage of caregivers believing children should be given meat at 6-8 months and continued breastfeeding at one years or above did not significantly change (*P* = 0.7223 and *P* = 0.1711 for trend, respectively), remaining around 50% and 20% respectively in the four surveys.

### Sources of infant feeding information

**Figure 3:**
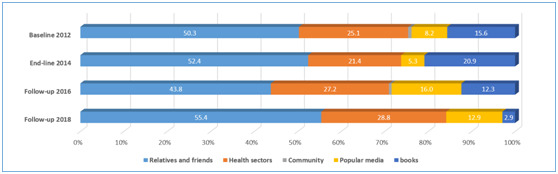
Source of caregivers' information on complementary feeding.

**Figure 3** shows the sources of caregivers’ information on complementary feeding in the four surveys. Around half of the caregivers got the information of complementary feeding from their relatives and friends and only about 25% from health sectors. The proportion of information received through books in the four surveys declined, while the proportion of information received through popular media (eg, the Internet, radio and television, mobile, etc.) increased. There were very few caregivers who received complementary feeding information from the community (less than 1%), which included midwives and family planning workers.

## DISCUSSION

During the trial period from 2012 to 2014, two core complementary feeding practices indicators, minimum dietary diversity and introduction of (semi-) solid food foods at 6-8 months, significantly increased in Huzhu County, however, there was no significant upward or even downward trend after the trial. Meanwhile, the prevalence of meal frequency and minimum acceptable diet were nearly unchanged and still suboptimal, maintaining around 30% and 10%, respectively from 2012 to 2018. Caregivers’ feeding knowledge was suboptimal as well. Although the proportion of caregivers who knew the duration of exclusive breastfeeding and introduction of semi or solid foods at 6-8 months increased during the period of six years, there were still around half of the caregivers who did not know the feeding recommendations. Caregivers in Huzhu County got information on complementary feeding mainly from their relatives and friends with only about 25% from health sectors, which was consist with previous studies in rural China [21-23].

Sustainability is one of the key issues of health programs, which is as important as the effectiveness of interventions to funders, program managers and policy makers [24]. Public health programs can only deliver benefits if they are able to sustain activities over time [25]. Financial resources may only be promised from a particular funder for a short period of time, however, how to keep public health programs sustained after then is of great concern [25]. In our study, we carried out two follow up assessments; one and a half years and four years respectively after the controlled intervention trial study ended in the intervention county, and showed that effectiveness of the intervention on minimum dietary diversity and introduction of (semi-) solid foods at 6-8 months was not maintain after the trial ended, which suggests that sustainability issues need to be addressed.

First, there is a need to strengthen the quality of infant and young child feeding (IYCF) counselling by village doctors. Village doctors in China are an older group than other health care doctors and often inadequately trained [26]. In our study county, the intervention was implemented through the rural three-tier health care system (county-township-village). Village doctors, as the main delivery channel, were trained to provide face-to-face complementary feeding counseling to caregivers as well as the distribution and explanation of YYB [14,15]. We conducted two quality IYCF trainings among village doctors in the study county in March 2013 and June 2014 respectively during the trial period, which has improved two core complementary feeding practices indicators [14,15]. However when the trial ended, lack of refresher training and supervision of village doctors may lowered the quality of IYCF counselling. Furthermore, the distribution of YYB provided no allowance yet increased the workload [15], therefore, village doctors may have been unwilling to and have had no time to conduct quality IYCF counselling. The results of our surveys showed that only about 25% of caregivers received feeding information from health sectors, which suggested that feeding counseling delivered by village doctors was not satisfactory. Therefore, improvement of the quality of IYCF counselling by village doctors is also greatly needed.

Community-based infant feeding promotion and support has been proven to be effective on breastfeeding and complementary feeding practices in other countries [27,28]. However, in our study county, very few caregivers received infant feeding knowledge from the community (less than 1%). In China, there is a large number of family planning workers in communities who are in charge of providing sexual and reproductive health services to unmarried young people [29]. In 2013, the Ministry of Health and the National Family Planning Commission were merged in China [30], and the family planning workers can also provide maternal and child health service in rural areas. Therefore, community family planning workers could be mobilized to deliver the IYCF counselling as well.

The information-motivation-behavior skills (IMB) model indicates that information can be transformed into action that can motivate individuals and eventually influence their attitudes and behavior [31,32]. With the development of information technology and social media, smartphones have become new channels for information acquisition, and these are widely used in health education research [33-35]. With the socio-economic development and the improvement of people's living conditions in China, mobile phones and the Internet have spread to millions of households in China. Data showed that, by the end of 2017, there were 1417 million mobile phone users and 772 million Internet users, of which 753 million were smartphone Internet users. The Internet penetration rate reached 55.8%, of which 35.4% was in rural areas [17]. An individually randomized controlled trial found that health education through WeChat (the most popular social media app in China) achieved greater improvement in malaria-related knowledge, attitude, practice, and skills. Furthermore, participants had high levels of satisfaction with WeChat health education and more than 80% of them would continue to follow the WeChat account [17]. A systematic review showed the feasibility of delivering eHealth interventions to improve health literacy skills among people with different health conditions, risk factors and socio-economic backgrounds [36]. Our surveys showed that about 70% of the mother's education was in junior high school or above (9 years or more of education). The popularity of smartphones and the Internet, and the education level of the younger generation of mother provide a foundation for disseminating feeding recommendations directly to mothers through mobile phones and the Internet. Our team conducted a randomized controlled trial from 2019 to 2020 by using a WeChat account to promote breastfeeding practices in Huzhu County, which proved WeChat breastfeeding education could improve the exclusive breastfeeding rate in the early postnatal period [37]. Further studies are needed to assess the effectiveness of WeChat accounts on improving the complementary feeding practice in rural areas in China.

Improving complementary feeding is particularly challenging globally because of the multiple complex behaviors involved [38]. Previous study showed that training, monitoring, and evaluation declined in frequency and quality, or were eliminate altogether which influenced the Sustainability of the Alive & Thrive initiative (A&T) in Bangladesh and Vietnam [39], which was in accordance with our findings. Regular monitoring along with process and impact evaluations feed information back to programs is necessary [38]. Therefore, our study carried out a routine surveillance of the complementary feeding program, and was an important example for other similar program.

The strength of our study is that we continuously collected data on infant feeding practices and caregivers’ feeding knowledge by using the same methods and standards in the same county. Therefore, we obtained the trends of infant feeding practices and feeding knowledge in order to assess the sustainability of the program. However, our study also has limitations. First, the sample size was smaller at the two follow-ups than at the baseline and end-line surveys, which might lead to bias. Second, we just evaluated the program in only one county, thus a generalization of the results should be done with caution.

## CONCLUSIONS

Based on analysis of the trends in complementary feeding practices and feeding knowledge from 2012 to 2018, this study showed that the sustainability of the community-based YYB and dietary counseling program was suboptimal. We suggest that multiple information delivery channels such as smartphones and the Internet should be explored as a supplement to existing information channels for delivering counseling. Future programs need to monitor program implementation in other settings in China and elsewhere.
